# Exercise stereotypes and fatigue in people living with HIV: does self-efficacy play a mediating or a moderating role?

**DOI:** 10.1186/s13690-018-0269-1

**Published:** 2018-04-30

**Authors:** Laura Gray, Aïna Chalabaev, Jacques Durant, Eric Rosenthal, Christian Pradier, Martin Duracinsky, Isabelle Rouanet, Laura Schuft, Serge S. Colson, Fabienne d’Arripe-Longueville

**Affiliations:** 1 Faculté des Sciences du Sport, Université Côte d’Azur Laboratoire Motricité Humaine, Expertise, Sport, Santé (LAMHESS – EA 6312), 261 Boulevard du Mercantour, 06203, Cedex 3 Nice, France; 2 Univ. Grenoble Alpes, SENS, F-38041 Grenoble, France; 3 Department of Infectious Diseases, Université Côte d’Azur, CHU, Archet 1, France; 4 Department of Public Health, Université Côte d’Azur, CHU, Archet 1, France; 5 AP-HP Department of Clinical Research Bicêtre Hospital, (Le Kremlin Bicêtre), France; 6 Department of Infectious Diseases Nîmes Hospital, (Nîmes), France

## Abstract

**Background:**

Recent research suggests that exercise stereotypes may influence physical activity through ego depletion and internalization mechanisms. The objective of this study was to better understand exercise stereotypes mechanisms among people living with HIV (PLHIV) by further examining the role of exercise self-efficacy and perceived physical fatigue in the relationship between exercise stereotypes and physical activity.

**Methods:**

Three hundred five people living with HIV were recruited to provide data on their stereotypes related to exercise, exercise self-efficacy, perceived physical fatigue as well as their level of physical activity (PA).

**Results:**

From the different models tested, the serial mediation model with exercise self-efficacy and perceived physical fatigue as mediators of the relationship between exercise stereotypes and PA, as well as the moderated mediation model with exercise selfefficacy as a moderator of exercise stereotypes and perceived physical fatigue a mediator, provided good fits to the data. However, the moderated mediation model (with indirect associations between negative exercise stereotypes and PA via perceived physical fatigue being moderated by exercise self-efficacy) explained the most variance in PA (R2 = .27).

**Conclusion:**

The moderated mediation model suggests that exercise stereotypes might influence PA through ego depletion mechanisms and be tempered by exercise self-efficacy.

## Background

Stereotypes are defined as shared beliefs concerning personal characteristics and behaviors of a group of persons [1]. Researchers have identified numerous pathways through which stereotypes impact health behaviors, primarily through internalization into the self, stereotype threat, downward social comparison/resilience, stereotype boost and upward social comparison/role models [2]. For example, according to the Stereotype Embodiment Theory (SET) [3], in older adults the relationships between aging stereotypes and health outcomes can be explained by the stereotype internalization hypothesis. This suggests that age stereotypes are internalized into self-perceptions of aging, and aging experiences are interpreted via this process [4]. Older adults who have negative perceptions of their aging consider that health problems are inevitable consequences of aging. This, in turn, could lead older adults to view health behaviors as futile [5] and hinder self-efficacy [6]. Indeed, self-efficacy was a potential mediator in the relationship between aging stereotypes and task performance [6]. In contrast positive views of aging may enhance adoption of preventive health behaviors in older adults entailing that the higher positive self-perceptions of aging the more older adults engage in healthy practices as opposed to those with more negative aging self-perceptions [5].

Physical activity (PA) is one of the most important health behaviors associated with prevention and management of chronic diseases, and stereotypes appear as an important factor of this behavior. For example, Wurm, Tomasik, and Tesch-Romer [7] showed that positive views of aging contributed to a higher level of PA. Besides age stereotypes, exercise stereotypes have been identified in recent literature, as playing a major role in the relation to PA [8, 9]. These exercise stereotypes, which different populations endorse, or adhere to, to varying degrees, can be both positive and negative and have been the source of specific exercise stereotype scales being developed for the elderly [10], cancer patients [8], and more recently for people affected by HIV/AIDS [9]. These scales allowed to identify two categories of stereotypes. The positive stereotypes category included shared beliefs about exercise benefits (e.g., “Exercise improves the morale of HIV-infected patients”). The negative exercise stereotypes category was composed of shared beliefs about lack of capacity for exercise (e.g., “HIV-infected patients do not have enough physical resources to exercise”), exercise risks (e.g., “Exercise should be avoided by HIV-infected patients because it causes injuries”), and treatment side effects (e.g., “Because of treatments, HIV-infected patients do not have enough energy to exercise”).

In order to develop effective interventions, it is crucial to identify the mechanisms through which exercise stereotypes influence PA among people viewed as vulnerable such as the elderly or people living with chronic disease. A first mechanism, through which exercise stereotypes would influence PA, is stereotype internalization. In their cross-sectional study, Emile et al. [11] reported that exercise stereotypes were related to older adults’ engagement in PA through physical self-worth, suggesting such an internalization process. In a further longitudinal study, Emile et al. [12] emphasized the importance of distinguishing between general self-perceptions and more domain-specific perceptions indexed by physical self-worth and the role of this physical self-worth in health behaviors. While the stereotype internalization mechanism was supported in their study, the results revealed a direct relationship between exercise stereotype endorsement and adoption of an active lifestyle, independently of how people perceived their own aging. Thus, these results suggested that self-perceptions of aging, the active construct in internalization, may not be the only pathway through which stereotype endorsement may affect older adults’ health-related behaviors.

Another pathway through which exercise stereotypes could affect PA and health-related outcomes is ego depletion. Ego depletion corresponds to the idea that engaging in acts of self-control (i.e., overriding initial impulses or responses such as resting on a couch to watch TV to engage in regular exercise) draws from a limited “reservoir” which, when depleted, results in reduced capacity for further self-regulation [13]. Negative stereotypes have been shown to reduce self-control and affect one’s ability to self-regulate [14] because coping with such stereotypes requires mental energy, reducing in turn subjective vitality (i.e., the energy one can harness or regulate for purposive actions) or increasing perceived fatigue (i.e., fatigue that is consciously felt). Likewise, Emile et al. [12] reported that the endorsement of negative exercise stereotypes in older adults negatively predicted subjective vitality, suggesting that ego depletion could be a pathway through which exercise stereotypes affect older adults’ health, complementing the internalization pathway. In the same way, a meta-analysis by Hagger, Wood, Stiff & Chatzisarantis [15] showed a significant effect with medium to large magnitudes of exerting self-control on acute subjective or perceived fatigue.

In sum, the recent literature suggests that exercise stereotypes could influence health behaviors including PA through different pathways or mechanisms: (a) stereotype internalization and (b) ego depletion. Yet, it is necessary to further elucidate the relationships and/or role played by each mechanism. Our study takes a closer look at internalization and ego depletion mechanisms in people living with HIV (PLHIV). This population was selected as fatigue is one of the most reported recurrent symptoms with a prevalence rate varying from 33% to 88% [16, 17]. In addition PLHIV do not engage enough in regular PA according to World Health Organization standards [18, 19]. Although some possible reasons are shared by the general population (e.g., lack of interest; lack of time), other reasons might be more specifically related to chronic illness, such as the endorsement of negative exercise stereotypes acting as psychological barriers. In line with previous studies having considered self-perceptions as an index of stereotypes internalization in vulnerable populations [5, 11, 12, 20], we looked at exercise self-efficacy, defined as an individual’s belief in his or her ability to succeed in a specific situation to accomplish a task [21], as a situational indicator of the internalization mechanism. In addition, based on Hagger et al. [15], we considered perceived physical fatigue as an index of the ego depletion mechanism. Based on existing literature, this study argues that exercise self-efficacy and perceived physical fatigue would mediate relationships between exercise stereotypes and PA variables in PLHIV, and aims at determining the nature of this mediation. For this purpose, several competing mediation models were tested.

## Methods

### Study design

A quantitative cross-sectional study design was employed from 1st of September 2016 to 30th of June 2017 in hospitals in three populated regions of France including the capitol.

### Participants

A sample of 344 men (*n* = 245) and women (*n* = 99) living with HIV participated in this study. The staff of the three French hospitals’ departments of infectious diseases screened and assessed patients for eligibility. Participants were included in the study if they were between 40 and 65 years old, were under treatment for at least 10 years and were followed by doctors of one of the three hospitals. As fatigue seems to be more common among co-infected individuals [22], we did not include PLHIV affected by other diseases such as hepatitis C, cancer, renal dysfunction and sclerosis; for the same reason pregnant women were excluded. The participants could read and understand the questionnaires of the survey written in French, and they were not affected by cognitive impairment. The response rate was 54% ± 9%. After data cleaning, a complete analytic sample of *n* = 305 (men, *n* = 228; women, *n* = 77; M = 53.5; SD = 9.58) was retained for statistical analysis. All participants were informed that the survey was voluntary and that all responses would remain anonymous and confidential. The study complied with national ethical standards (n°CNIL/UNS/2015/0007).

### Procedure

The survey was distributed by staff from the virology services of the three participating hospitals. The staff member presented the study objectives and project partners and assured the anonymity of participants. A room was made available by the hospital for completing the questionnaires. The staff member remained in the room to respond to any requests for clarification. The average time of completion was 20 min.

### Measures

#### HIV exercise stereotypes

We used the HIV Exercise Stereotype Scale (HIVESS) developed and validated by Gray et al. [9] to assess endorsement of exercise stereotypes in PLHIV. The HIVESS consists of 14 items divided into three subscales: (a) five items measured stereotypes related to exercise benefits for PLHIV (e.g., “Physical activity improves the morale of HIV-infected patients”), (b) four items measured stereotypes related to exercise risks linked to injury (e.g., “Practicing a physical activity should be avoided by HIV-infected patients because it causes injuries”) and linked to contamination (e.g., “HIV-infected patients do not practice physical activities because they could contaminate someone during the activity”) and (c) five items measured stereotypes related to a lack of capacity of PLHIV for exercise (e.g., “HIV-infected patients do not have enough physical resources to practice a physical activity”). Participants answered on a 6-point Likert scale ranging from 1 (“do not agree at all”) to 6 (“totally agree”).

#### Perceived physical fatigue

We used the physical fatigue sub-scale of the Fatigue Intensity Scale (FIS) [23], a valid scale previously used in French individuals [24, 25]. An example item was: “I have trouble maintaining physical effort for long periods”. Participants responded on a 6-point Likert scale ranging from 1 (“do not agree at all”) to 6 (“totally agree”). This subscale was selected because it is specifically related to physical fatigue characteristics as opposed to other used scales such as the HIV-Related Fatigue Scale [26] in which the characteristics are confounded with their consequences, circumstances and triggers.

#### Exercise self-efficacy

Exercise self-efficacy was measured using six items adapted from Bandura et al. [21, 27] guidelines (e.g., “I can be physically active regularly”). Participants answered on a 6-point Likert scale ranging from 1 (“do not agree at all”) to 6 (“totally agree”).

#### Physical activity

We used the Dijon Physical Activity Score [28], developed and validated to measure PA level. This scale contains 9 items and assesses participants’ level of PA via: (a) an overall appraisal of one’s PA (“Do you consider yourself to be physically: from (1) very active and athletic, to (4) completely sedentary?”); (b) two items on everyday activities (“On a weekly basis, your everyday activities take you: from (1) more than 10 hours, to (5) no time spent”); (c) five items on sport and leisure activities (e.g., “For how many months of the year do you engage in these activities (sport or leisure)?”); and (d) one item on rest (“On a daily basis, you rest (sleep, nap or wakeful rest): from (1) less than 12 hours, to (4) more than 20 hours”). The scores on each item are totaled, with the total score out of 30 points indicating a participant’s level of PA (individuals who score below 18 being considered as inactive). This measure, which has been shown to be a reproducible and valid measure of PA both among older adults and among patients with coronary artery disease [29], was deemed relevant to our population with chronic illness.

#### Covariates

Age and previous level of physical activity were the covariates assessed in this study. These variables were chosen based on validated previous research suggesting that they are related to PA participation. Studies on older adults have revealed age differences in health related behaviors [20] and recently in PA participation [30]. Furthermore, evidence reveals an association between participation in PA in later years and earlier PA behaviors, previous participation in PA positively predicting future engagement [31, 32].

#### Age

Age was recorded in a standard manner with the following item: “Thank you for stating your date of birth (dd/mm/yyyy)”.

#### Previous level of physical activity

Previous level of physical activity (PPA) was assessed with a question asking to consider one’s level of physical activity, including daily PA such as leisure or competitive sports and active commuting (i.e., walking or cycling to work), before being diagnosed as HIV-positive [31]. This question was stated as follows: This question is related to your physical activity before the condition. “You consider yourself as having been…”. Participants were then asked to rate their answer on a Likert scale from 1 “very sportive/physically active” to 6 “not physically active at all”.

#### Data analyses

Data analyses were performed using SPSS.22 and AMOS 20.0. The analysis allowed for missing values to be replaced by using multiple imputation [33]. Statistical analyses included several methods. Descriptive analyses (i.e., mean, standard deviation) were run and the reliability of the questionnaires was assessed with Cronbach’s alpha indicating internal consistency. The significance of the relationships between all variables was calculated using Pearson bivariate correlations. To determine the variables significantly contributing to PA variance a stepwise regression analysis was computed. Then three competing mediation models were tested with SEM using AMOS 20.0. Three indexes were selected to express model fit: chi-square (χ^2^), the Root-Mean Square Error of Approximation (RMSEA), the Bentler Comparative Fit Index (CFI), Tucker Lewis Index (TLI) and the Normed Fit index (NFI). RMSEA values ≤0.08 at 90% Confidence Interval (RMSEA CI 90%) in combination with values for CFI, TLI and NFI ≥ 0.90 suggest an acceptable model fit. The hypothesized mediation effects of perceived physical fatigue and exercise self-efficacy were tested by mediation analyses following the bootstrap procedure recommendations proposed by MacKinnon and colleagues [34]. The bootstrap procedure resampled the data 5000 times and calculated the indirect effect for each sample in this study. The bias corrected 95% confidence intervals (CI) indicated significant or non-significant indirect effects when they did not contain zero.

**Fig. 1:**
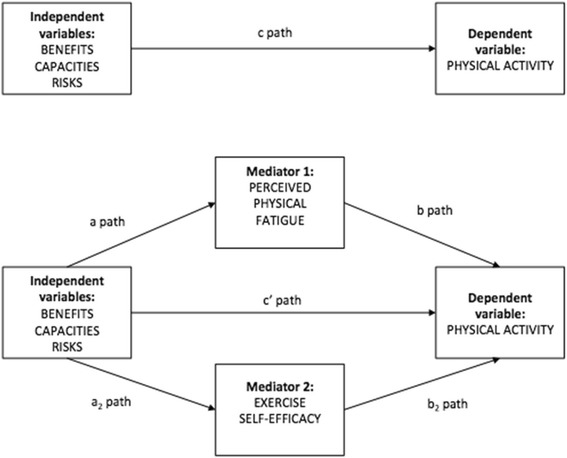
Descriptive parallel mediation model with indirect and direct pathways of exercise stereotypes dimensions on physical activity with perceived physical fatigue and exercise self-efficacy as mediators. Notes: *N* = 305; Benefits: exercise benefits stereotypes (IV); Capacities: lack of capacity for exercise stereotypes (IV); Risks: exercise risks stereotypes (IV); Exercise self-efficacy (mediator); Perceived fatigue (mediator); Physical activity (DV); a path: relationship between the independent variables and each mediator; b path: relationship between each mediator and the dependent variable; c path: relationship between the independent variables and the dependent variable (direct pathway); c' path: relationship between the independent variables and the dependent variable controlling for the mediators (indirect pathway)

The first model tested was a parallel mediation model in which the independent variables (IV) were the three dimensions of exercise stereotypes (i.e., exercise benefits; exercise risks; lack of capacity for exercise), exercise self-efficacy and perceived physical fatigue were the mediators, and level of PA was the dependent variable (DV) (see Fig. 1).

**Fig. 2:**
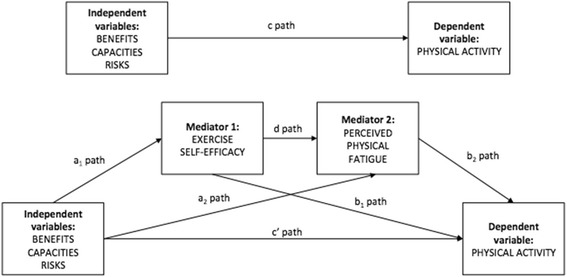
Descriptive serial mediation model with indirect and direct pathways of exercise stereotypes dimensions on physical activity with exercise self-efficacy and perceived physical fatigue as a mediators. Notes: *N* = 305; Benefits: exercise benefits stereotypes (IV); Capacities: lack of capacity for exercise stereotypes (IV); Risks: exercise risks stereotypes (IV); Exercise self-efficacy (mediator); Perceived fatigue (mediator); Physical activity (DV); a path: relationship between the independent variables and each mediator; b path: relationship between each mediator and the dependent variable; c path: relationship between the independent variables and the dependent variable (direct pathway); c' path: relationship between the independent variables and the dependent variable controlling for the mediators (indirect pathway); d path: influence of mediator 1 on mediator 2

The second model tested was a sequential mediation model in which constructs function as a causal chain. Exercise stereotypes (IV) would hereby predict perceived self-efficacy (first mediator), which would in turn predict perceived physical fatigue (second mediator) with an overall effect on level of PA (DV) (see Fig. 2).

**Fig. 3:**
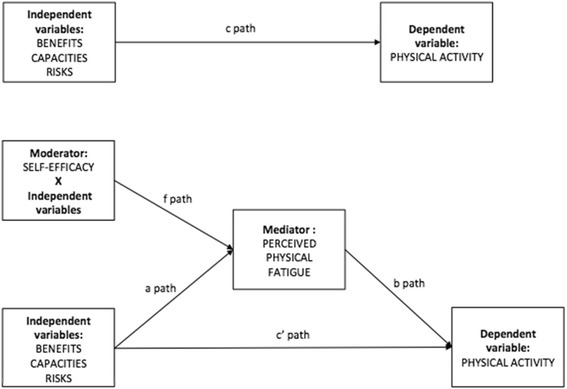
Descriptive moderated mediation model for exercise stereotypes dimensions with perceived physical fatigue as a mediator and exercise self-efficacy as a moderator. Notes: N = 305; Benefits: exercise benefits stereotypes (IV); Capacities: lack of capacity for exercise stereotypes (IV); Risks: exercise risks stereotypes (IV); Exercise self-efficacy (mediator); Perceived fatigue (mediator); Physical activity (DV); a path: relationship between the independent variables and each mediator; b path: relationship between each mediator and the dependent variable; c path: relationship between the independent variables and the dependent variable (direct pathway); c' path: relationship between the independent variables and the dependent variable controlling for the mediators (indirect pathway); f path: moderating variable on the interaction between the independent variables and the mediator

The third model tested examined whether the mediation of the exercise stereotypes – PA relationship by perceived physical fatigue was moderated by self-efficacy. Here the IV remained exercise stereotypes, with perceived physical fatigue as a mediator and exercise self-efficacy as a moderator and level of PA the DV (see Fig. 3).

Moderated mediation was run following recommendations from Muller et al. [35], suggesting the assessment of the overall treatment effect of the independent variable (i.e., exercise stereotypes) on the outcome variable (i.e., physical activity level). This was followed by the assessment of the moderation of the treatment effect of the independent variable on the mediator (i.e., perceived physical fatigue). Lastly, the moderation effect of the mediator on the outcome variable, as well as the moderation of the residual treatment effect (i.e., the direct effect of the independent variable controlling for the mediator) of the independent variable on the outcome variable, were assessed.

## Results

Results are presented in three parts. First, we present descriptive analyses. Second, we describe the results from the stepwise regression analysis. Third, we present the structural equation modeling analyses by looking at the results of the three hypothesized models.

### Descriptive analyses

Descriptive statistics, means and standard deviations; alpha coefficients, and bivariate correlations for all study variables are presented in Table 1.

**Table 1:** Means, Standard Deviations, Coefficient Alphas and Bivariate Correlations between exercise stereotypes dimensions, self-efficacy, perceived physical fatigue and physical activity in people living with HIV, France, 2017

	BEN	RIS	LCE	SE	FAT	PA	M	SD	α
BEN	1						4.60	1.14	.78
RIS	−.01 .86	1					1.90	1.11	.79
LCE	−.034 .55	.27^**^ .001	1				3.07	1.28	.84
SE	.40^**^ .001	−.18^**^ .001	−.29^**^ .001	1			4.84	1.37	.95
FAT	−.17^**^.001	.26^**^ .001	.54^**^ .001	−.44^**^ .001	1		3.21	1.54	.86
PA	.23^**^ .001	−.13^*^ .02	−.20^**^ .001	.46^**^ .001	−.27^**^ .001	1	19.7	5.17	–

*Notes*: *N* = 305, *FAT* Perceived physical fatigue, *BEN* Exercise benefits stereotypes, *RIS* Exercise risks stereotypes, *LCE* Lack of capacity for exercise stereotypes, *SE* Self-efficacy, *PA* level of physical activity, *M* Mean, *SD* Standard deviation, α Cronbach’s alphas

*p < .05. **p < .01, ***p < .001

### Regression analysis

A four-step hierarchical regression analysis identified the predictors of PA level as the dependent variable. In the first step, previous PA level (PPA) and age (AGE) (β = .33, *p* < .001; β = −.03, *p* = NS) significantly predicted PA level, R^2^ = .11. In the second step, when exercise stereotypes dimensions were added, exercise benefits (BEN, β = .15, *p* < .01), exercise risks (β = −.08, *p* = NS) and lack of capacity for exercise (β = −.19, *p* < .001) significantly predicted PA level, R^2^ = .18 (ΔR^2^ = .07). Perceived physical fatigue was entered in the third step and significantly predicted PA level, R^2^ = .20 (ΔR^2^ = .02). In the last step, exercise self-efficacy was added and significantly increased variance by 45% revealing the strongest prediction of PA level (ΔR^2^ = .09), Fchange (7, 305) = 35.77, *p* < 0.001. The four steps of the hierarchical regression determining the predictors of PA level are illustrated in Table 2.

**Table 2:** Stepwise regression analyses: Psychosocial predictors of physical activity in people living with HIV, France 2017

Steps	Predictors	F	df	R^2^	ΔR^2^	β	t
1	PPA	17.89***	2	.11		.33***	5.98
	AGE					−.03	−.46
2		9.17***	5	.18	.07		
	PPA					.32***	5.89
	AGE					.002	.04
	BEN					.15**	2.89
	RIS					−.08	−1.44
	LCE					−.19***	−3.53
3		6.31**	6	.20	.02		
	PAA					.30***	5.71
	AGE					.01	.17
	BEN					.13*	2.46
	RIS					−.06	−1.23
	LCE					−.12	−1.82
	FAT					−.16*	−2.51
4		35.77***	7	.29	.09		
	PPA					.28***	5.50
	AGE					−.01	−.90
	BEN					.02	.36
	RIS					−.03	−.63
	LCE					−.08	−1.41
	FAT					−.05	−.80
	SE					.35***	5.98

*Notes*. *N* = 305, *BEN* exercise benefits stereotypes, *RIS* exercise risks stereotypes, *LCE* lack of capacity for exercise stereotypes, *SE* exercise self-efficacy, *FAT* perceived physical fatigue, *F* Fisher-Snedecor distribution, *df* degrees of freedom, *R*^*2*^ standardized variance, *ΔR*^*2*^ delta R-squared or the change in R-squared between two equations, *β* beta weight values, t t-statistic values

*p < .05. **p < .01, ***p < .001

### Main analyses: model fit

Three hypothetical models were assessed in order to examine the role of exercise self-efficacy and perceived physical fatigue in the relationship between exercise stereotypes and PA.

### Parallel mediation model

The parallel model placing perceived physical fatigue and exercise self-efficacy on the same level (Fig. 1) and controlling for PA level before HIV infection as well as age did not provide a good model fit: χ^2^(8) = 36.076, *p* < .001, CFI = .918, TLI = .712, NFI = .902 RMSEA = .108 (90% confidence interval [CI] [.073, .144]).

### Serial mediation model

**Fig. 4:**
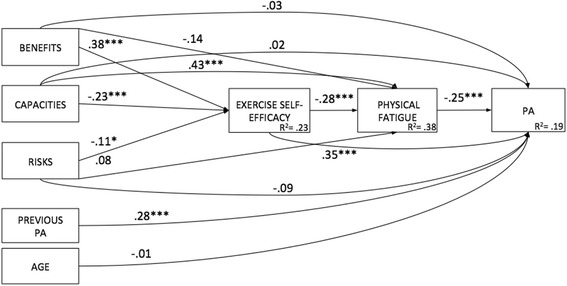
Serial mediation model with indirect and direct effects of exercise stereotypes dimensions on physical activity in people living with HIV, France 2017 Notes: N = 305; Benefits: exercise benefits stereotypes (IV); Capacities: lack of capacity for exercise stereotypes (IV); Risks: exercise risks stereotypes (IV); Exercise self-efficacy (mediator); Perceived fatigue (mediator); Previous PA: PA level before HIV status (covariate); Age (covariate); PA: physical activity level (DV); R^2^: standardized variance; *p* < .05 *; *p* < .01 **; *p* < .001 ***

The serial mediation model in which the first mediator was exercise self-efficacy and the second was perceived physical fatigue, controlling for PA level before HIV infection and age, provided a good fit to the data: χ^2^(7) = 8.59, *p* = .283, CFI = .995, TLI = .981, NFI = .977, RMSEA = .027 (90% confidence interval [CI] [.000, .079]) (see Fig. 4).

After controlling for covariates (age, previous level of PA), mediation analyses for the serial model revealed that the indirect effects of exercise stereotypes on PA were significant. No significant direct effects of exercise stereotypes on PA were observed (see Table 3). Specifically, exercise self-efficacy and perceived physical fatigue fully mediated the relationship between the different stereotypes (exercise benefits, exercise risks and lack of capacity for exercise) and PA accounting for 19% of variance in PA level.

**Table 3:** Total, direct and indirect effects of exercise stereotypes dimensions as independent variables and exercise self-efficacy and perceived physical fatigue as mediators on physical activity in people living with HIV, France 2017

	Mediators	IV → mediator (s)	Mediator(s) → DV	Total effect	Direct effect	Indirect effect	95% CI
BEN → PA	SE-FAT	.38***/−.05	.001**/−.16	.16**	.13	.02**	[.006 to .061]
RIS → PA	SE-FAT	−.11/−.08	.001**/−.16	−.08	−.06	−.02*	[−.051 to −.002]
LCE → PA	SE-FAT	−.23/.44	.001**/−.16	−.19**	−.12*	−.08*	[−.154 to −.018]

*Notes*. *N* = 305, *IV* independent variable, *DV* dependent variable, *BEN* exercise benefits stereotypes, *RIS* exercise risks stereotypes, *LCE* lack of capacity for exercise stereotypes, *SE* exercise self-efficacy, *FAT* perceived physical fatigue, *95% CI* lower and upper bound of bias-corrected 95% confidence interval with 5000 bootstrap samples

*p < .05. **p < .01, ***p < .001

### Moderated mediation model

**Fig. 5:**
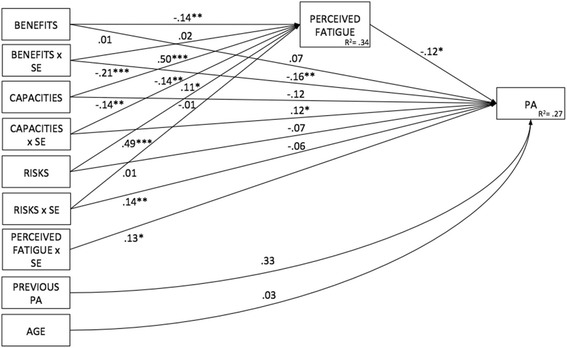
Moderated mediated results for exercise stereotypes dimensions and perceived physical fatigue across levels of exercise self-efficacy in people living with HIV, France 2017. Notes: *N* = 305; Benefits: exercise benefits stereotypes (IV); Capacities: lack of capacity for exercise stereotypes (IV); Risks: exercise risks stereotypes (IV); SE: exercise self-efficacy (moderator); Perceived fatigue (mediator); Previous PA: Pa level before HIV status (covariate); Age (covariate); PA: physical activity level (DV); R^2^: standardized variance; *p* < .05 *; *p* < .01 **; *p* < .001 ***

The third model examined whether the mediation of the exercise stereotypes – PA relationship by perceived physical fatigue was moderated by self-efficacy. A good fit was revealed, with the moderator: χ^2^(3) = 3.265, *p* = .353, CFI = .999, TLI = .988, NFI = .993, RMSEA = .017 (90% confidence interval [CI] [.000, .100]) (see Fig. 5).

We then followed the steps outlined by Muller et al. [35] to test for mediated moderation effect. To this purpose, parameters for three regression equations were estimated to assess: (a) the overall treatment effect of exercise stereotypes on PA level as moderated by exercise self-efficacy, (b) the treatment effect of exercise stereotypes on perceived physical fatigue as the mediator moderated by exercise self-efficacy and (c) the mediator’s (partial) effect on the outcome variable PA and the residual effect of the treatment (exercise stereotypes) on PA, controlling for the mediator (perceived physical fatigue) moderated by exercise self-efficacy (see Table 4). The first equation (a) revealed significant direct effects of exercise benefits on PA and of exercise self-efficacy on PA. The second equation (b) revealed a significant effect of lack of capacity for exercise stereotype on perceived physical fatigue that was moderated by exercise self-efficacy (b = −.06, *p* < .01). The third equation (c) showed that the partial effect of perceived physical fatigue on PA was not significant but a significant interaction effect between self-efficacy and lack of capacity stereotype on PA was observed (b = .02, *p* < .05). This model predicted 27% of variance in PA (see Fig. 2).

**Table 4:** Total, direct and indirect effects of exercise stereotypes dimensions as independent variables, perceived physical fatigue as mediator and exercise self-efficacy as a moderator on physical activity in people living with HIV, France 2017

	Mediators	IV → mediator(s)	Mediator(s) → DV	Total effect	Direct effect	Indirect effect	95% CI
BEN → PA	FAT	.07**	−.12	.09*	.07	.02*	[.002to .040]
BENxSE→PA	FAT	−.16	−.12	−.16**	−.16**	−.003	−.017 to .004]
RIS → PA	FAT	.001*	−.12	−.09	−.07	−.01*	[−.036 to −.001]
RISxSE→PA	FAT	−.06	−.12	−.06	−.06	.001	[−.009 to .018]
LCE → PA	FAT	.001**	−.12	−.17**	−.11	−.06	[−.116 to −.007]
LCExSE→PA	FAT	−.06**	−.12	.14*	.12	.02*	[.002 to .037]

*Notes*. *N* = 305, *IV* independent variable, *DV* dependent variable, *BEN* exercise benefits stereotypes, *RIS* exercise risks stereotypes, *LCE* lack of capacity for exercise stereotypes, *SE* exercise self-efficacy, *FAT* perceived physical fatigue, *95% CI* lower and upper bound of bias-corrected 95% confidence interval with 5000 bootstrap samples

*p < .05. **p < .01, ***p < .001

Simple slope analyses were then run following suggestions from Dawson [36] that plotting the effects allows to observe their size and precise nature making interpretation easier [36]. With the simple slopes we can know the direction of the relationship for high and low levels of the moderating variable [37]. In our analysis, we looked at the specific relationship between exercise stereotypes and level of PA at different levels of exercise self-efficacy. We wanted to know if, for example, for PLHIV with high exercise self-efficacy there is evidence that their exercise beliefs (stereotypes) would be beneficial for their level of PA by running simple slope tests as suggested by Cohen et al. [38]. Of course, we took into consideration that we have positive and negative exercise stereotypes and that high levels of exercise self-efficacy will interact differently with these stereotypes in the sense that high exercise self-efficacy would reduce the impact of negative exercise stereotypes whilst enhancing that of positive exercise stereotypes.

**Fig. 6:**
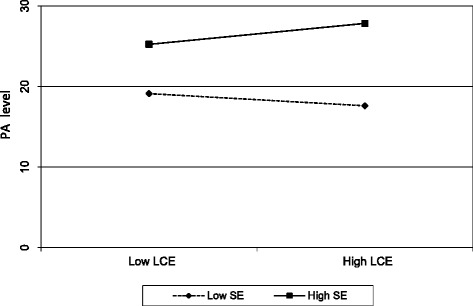
Simple slope analysis for lack of capacity for exercise at high and low levels of exercise self-efficacy for physical activity in people living with HIV, France 2017.Notes: *N* = 305. Functions are graphed for two levels of exercise self-efficacy: 1 standard deviation above the mean and 1 standard deviation below the mean. SE: exercise self-efficacy; LCE: lack of capacity for exercise stereotypes

Simple slope tests were run on the significant interaction effect of exercise stereotypes related to lack of capacity for exercise (*p* = .022) on PA level as the outcome, showing that lack of capacity for exercise varies as a function of exercise self-efficacy as the moderator (see Fig. 6). At low levels of self-efficacy (slope = −.804, *t* = − 2.54, *p* < .01) lack of capacity for exercise stereotypes was significantly negatively related to PA level. This suggests that the more participants endorsed stereotypes related to the lack of capacity for exercise, the less they were physically active. For high levels of self-efficacy (slope = .222, *t* = 1,56, *p* < .05), lack of capacity for exercise stereotypes was significantly positively related to PA level. Here, participants showed that lower levels of endorsement of stereotypes related to a lack of capacity for exercise corresponded to higher levels of PA.

## Discussion

This study examined the relationship between positive and negative exercise stereotypes and PA as a health behavior, focusing on the underlying psychological mechanisms among PLHIV. Different competing models were tested in order to examine the role of exercise self-efficacy and perceived physical fatigue in the relationship between exercise stereotypes and PA. As a brief overview, the serial mediation model (with exercise self-efficacy and perceived physical fatigue as mediators), and the moderated mediation model (with exercise self-efficacy as a moderator of exercise stereotypes and perceived physical fatigue a mediator) showed good fits. The moderated mediation model explained the most variance in PA (R^2^ = .27) with indirect links for exercise benefits and risks remaining significant. Lack of capacity for exercise was moderated by exercise self-efficacy for both high and low levels with significant effects on PA.

The serial mediation was run to observe the plausible causal chain in which the constructs function. This model was contingent to Levy and Bavishi’s [39] research, which showed that the C-reactive protein, a marker of increasing stress-related inflammation, played a mediating role in the relationship between self-perceptions and longevity. This suggests additional mediators between self-perceptions and observed health outcomes. Inflammation is a stress marker and inducing stereotypes causes stress (see [40]), which can be related to fatigue and be an indicator of the ego depletion mechanism [15]. The different exercise stereotypes (exercise benefits, exercise risks and lack of capacity for exercise) were indirectly associated to PA through exercise self-efficacy and perceived physical fatigue. Specifically, the higher the positive exercise stereotypes, the higher the exercise self-efficacy, the lower the perceived physical fatigue and the higher the level of PA, with the contrary being observed for negative exercise stereotypes. The overall model explained 19% of variance in PA level. This serial mediation of exercise self-efficacy and perceived physical fatigue confirmed the role of multiple mediators in the relationship between stereotypes and health outcomes such as PA. Our findings would extend the existing literature by showing that, beyond stress-related inflammation, perceived physical fatigue could be another valuable candidate in the relationship between self-perceptions and health outcomes.

The moderated mediation model placed self-efficacy as a moderator of exercise stereotypes in the causal chain: exercise stereotypes > perceived physical fatigue > PA level. This model was tested as previous studies have shown that people with higher self-perceptions in the physical domain have not internalized the stereotypes or have distanced themselves from their group and are thereby less sensitive to the stereotypes [30, 41, 42].

Results showed that indirect association between negative exercise stereotypes in terms of lack of capacity for exercise and PA via fatigue was fully moderated by exercise self-efficacy, explaining 27% of variance in PA level. For PLHIV with low levels of exercise self-efficacy, high negative exercise stereotypes are risk factors that increase their perceived physical fatigue, which in turn decreases their level of PA. In contrast, this negative chain is reduced in PLHIV with high levels of exercise self-efficacy. The moderated mediation model results are in line with previous studies that have evidenced that stereotypes can influence health outcomes through an ego depletion mechanism as operationalized through perceived fatigue in self-control task performance [15] and subjective vitality in the physical activity domain [12]. Furthermore, our results enrich this literature by showing that exercise self-efficacy could act as a moderator in this mechanism.

The parallel model that was tested revealed a near but non reliable fit, suggesting that a dual pathway, considering internalization [3] and ego depletion [12] side by side, does not best describe the mediating role of self-efficacy and perceived physical fatigue in the relationship between exercise stereotypes and PA level. However, the near fit for this model encourages the importance of each of these mechanisms and calls for further investigation as to how these mechanisms interact.

The two viable models provide support for multiple pathways of influence of positive and negative exercise stereotypes in PLHIV (i.e., internalization and ego depletion), while suggesting their differential effects on PA level. The parallel and serial mediation models are contingent with recent research (see [12]) providing support for the role of both internalization and ego depletion mechanisms in the relationship between exercise stereotypes and health outcomes such as PA. Moreover, results suggest that exercise self-efficacy would play a complex role in the relationship between exercise stereotypes and physical activity, playing either a mediating role or a moderating role. However, the higher explained variance of the moderated model (R^2^ = .27) suggests retaining the role of self-efficacy as moderator.

### Limitations and future directions for research and intervention

This study points out some limitations. The data being of correlational nature, inferences can not be made about causal relationships between exercise stereotypes, mediators (perceived physical fatigue and self-efficacy), moderators (self-efficacy) and patients self-reported level of PA. Also, temporal causality cannot be inferred as the relationship between perceived physical fatigue and self-efficacy as regards to PA level could be recursive with a lower level of PA causing higher perceived physical fatigue or less self-efficacy which, in turn, discourages PA participation. Furthermore, the sample in this study presents specific sociodemographic characteristics in terms of gender, socioeconomic status and types and frequency of PA, thus limiting the generalization of the results. Moreover, although the moderated mediation model explains a higher variance in PA, further research is still needed to clearly establish the role of exercise self-efficacy as a mediator or as a moderator.

Despite these limitations, this study allows for several important implications in public health. First, as HIV is increasingly considered a chronic illness for people who have access to combined antiretroviral therapy, PLHIV are living longer and are faced with the health-related consequences of HIV [43–45]. Exercise presents itself as a self-management strategy that can limit disability, including mental, cognitive, physical and emotional symptoms, and enhance quality of life [46]. Hereby, the better understanding of the role of psychological barriers related to exercise stereotypes provided by the present findings, is of great interest to promoting a physically active lifestyle among PLHIV. Second, and more specifically, the fact that positive exercise stereotypes are positively related to PA via exercise self-efficacy and perceived physical fatigue encourages the promotion of the benefits of PA among people living with HIV and health professionals through different communication strategies. Furthermore, exercise self-efficacy as a moderator of stereotypes related to lack of capacity for exercise points to the interest of positive individual PA experiences that nurture self-efficacy, through for example adapted physical activity offers and interventions [27]. Such perspectives however call for additional research based on the experimental manipulation of exercise self-efficacy and its effects on stereotype endorsement and health outcomes, and also call for longitudinal research to observe the effects of manipulated self-efficacy over time.

## Conclusion

The moderated mediation model emphasizes that exercise stereotypes in people living with HIV might influence their PA through the ego depletion mechanisms as indexed by perceived physical fatigue, and be tempered by exercise self-efficacy. However, the relatively low explained variance for PA calls for further research, namely in order to investigate other variables in the relationship between exercise stereotypes and PA. Furthermore, it is of interest to consider the double status of exercise self-efficacy (moderator and mediator) in the relationship between exercise stereotypes and PA, as these roles enrich existing literature in the mechanisms at play [12] and encourage complementary experimental investigations.

## Abbreviations

BEN: Exercise benefits
CFI: Comparative fit index
CI: Confidence interval
FAT: Perceived physical fatigue
HIV: Human immunodeficiency virus
LCE: Lack of capacity for exercise
NFI: Normed fit index
PA: Physical activity
PLHIV: People living with HIV
PPA: Previous physical activity
RIS: Exercise risks
RMSEA: Root mean square error of approximation
SE: Exercise self-efficacy
TLI: Tucker-Lewis index
